# Long-term prognostic comparison of surgery followed by adjuvant chemoradiotherapy versus definitive chemoradiotherapy in T4N0-3M0 esophageal squamous cell carcinoma: a single-center retrospective cohort study

**DOI:** 10.3389/fonc.2026.1743644

**Published:** 2026-03-24

**Authors:** Shiwang Wen, Hesong Wang, Xiaohan Zhao, Shuguang Li, Chunyang Song, Wenzhao Deng, Jingyuan Wen, Shuchai Zhu, Wenbin Shen

**Affiliations:** 1 Department of Thoracic Surgery, Fourth Hospital of Hebei Medical University, Shijiazhuang, Hebei, China; 2 Department of Radiation Oncology, Fourth Hospital of Hebei Medical University, Shijiazhuang, Hebei, China

**Keywords:** adjuvant chemoradiotherapy, definitive chemoradiotherapy, esophageal squamous cell carcinoma, surgery, T4N0-3M0 stage

## Abstract

**Background:**

This large retrospective study aimed to compare the long-term survival outcomes of esophageal squamous cell carcinoma (ESCC) patients with stage T4N0-3M0 treated with surgery followed by adjuvant chemoradiotherapy (S+CRT) versus definitive chemoradiotherapy (dCRT).

**Methods:**

Patients with T4N0-3M0 ESCC who received S+CRT or dCRT at our institution between January 2011 and December 2018 were included in the study.

**Results:**

A total of 490 patients with stage T4N0-3M0 ESCC met the enrollment criteria, with 108 in the S+CRT group and 382 in the dCRT group. Overall survival (OS) and progression-free survival (PFS) were significantly better in S+CRT group compared to the dCRT group (P = 0.000, 0.003). After propensity score matching (PSM) analysis, 138 patients in the dCRT group and 81 patients in the S+CRT group were successfully matched. OS and PFS were again significantly better in the S+CRT group compared to the dCRT group (P = 0.002, 0.019).

**Conclusions:**

S+CRT is more likely to improve the overall prognosis of patients with T4N0-3M0 ESCC compared to dCRT. Treatment plan should be made based on a thorough consideration of both the patient’s condition and tumor characteristics, leading to individualized decisions.

## 1 Background

More than 50% of new cases and deaths from esophageal cancer (EC) worldwide occur in China, with squamous cell carcinoma being the predominant pathological type (1). Due to the insidious onset and aggressive nature of EC, most patients present at advanced stage and are ineligible for direct surgical resection by the time they seek treatment (2). Since the publication of the Dutch CROSS study, neoadjuvant chemoradiotherapy (nCRT) followed by surgery (S) has been the primary recommended approach for patients with resectable, locally advanced esophageal cancer. However, patients with T4 stage cancer were not included in this study (3). Due to the complexity and heterogeneity of the clinicopathological characteristics of patients with stage T4 EC, their optimal treatment options remain a subject of active clinical debate. The NCCN guidelines recommend definitive chemoradiotherapy (dCRT) or nCRT-S for patients with stage T4a EC, while dCRT is recommended for patients with stage T4b EC (4). Several studies have shown that, compared to dCRT, the survival benefits of nCRT-S are primarily observed in patients who respond effectively to neoadjuvant treatment (5–8). Historically, there was limited medical evidence supporting the use of postoperative adjuvant chemoradiotherapy (POCRT) in patients with EC. However, multiple studies have demonstrated the effectiveness of POCRT in post-operative EC patients with deep local invasion and positive lymph nodes (9–11). As the standard treatment approach for patients with T4 stage ESCC remains unclear, comparative studies evaluating the effectiveness of surgery followed by adjuvant chemoradiotherapy (S+CRT) versus dCRT in T4 ESCC are currently limited. Therefore, we analyzed the long-term survival outcomes of patients with stage T4N0-3M0 ESCC who underwent S+CRT or dCRT over a 5-years period at a single center, aiming to provide guidance for the clinical management of stage T4N0-3M0 ESCC.

## 2 Methods

### 2.1 Inclusion and exclusion criteria

The study was conducted in accordance with the Declaration of Helsinki (2013 revision) and was approved by the Ethics Committee of the Fourth Hospital of Hebei Medical University (Approval No. 2024KY202). Individual informed consent was waived due to the retrospective nature of the study.

The inclusion criteria for this study were as follows: patients were required to have pathologically confirmed ESCC and a diagnosis of stage T4N0-3M0 based on the AJCC 8th edition pathological and clinical TNM staging criteria. Those who underwent surgery were required to receive POCRT within 1 to 3 months postoperatively. For patients receiving dCRT, chemotherapy and radiotherapy were required to be administered concurrently, followed by consolidation chemotherapy after the completion of radiotherapy. Additionally, all patients were required to have adequate functional reserves of vital organs, including the heart, kidneys, liver, lungs and bone marrow.

Exclusion criteria included patients with pathologically confirmed adenocarcinoma, small cell carcinoma, or other pathological types. Patients with distant metastasis confirmed by imaging or pathology, either before or after treatment, were also excluded. Surgical patients who underwent R2 resection were excluded, as were patients receiving palliative treatment, those who had undergone therapeutic neoadjuvant treatment, and individuals with incomplete data or follow-up records.

### 2.2 Staging standards

For patients receiving dCRT, the clinical TNM stage was assessed based on medical records, contrast-enhanced CT of the chest and abdomen, endoscopic ultrasonography (EUS), and/or positron emission tomography/computed tomography (PET/CT). For surgical patients, the TNM stage was primarily determined through postoperative pathology reports. All patients were independently evaluated by two researchers using the AJCC 8th edition TNM staging system to replace their treatment staging.

### 2.3 Treatment modality

Treatment allocation was determined by multidisciplinary team evaluation based on tumor resectability, performance status, comorbidities, and patient preference. Patients with clinical T4a disease, good ECOG performance status (0–1),and low surgical risk who were expected to achieve R0 resection were recommended for S+CRT. Patients with clinical T4b disease, poor general condition, severe comorbidities, or unresectable tumors were assigned to dCRT. Patients who preferred organ-preservation therapy were also treated with dCRT. Based on whether surgical treatment was performed, patients were divided into two groups:S+CRT and dCRT.

In the S+CRT group, transthoracic esophagectomy with three-field lymph node dissection via right chest, upper abdominal, and cervical incisions was the predominant surgical approach. Video-assisted thoracoscopic surgery (VATS) was performed in selected patients with favorable anatomy. All surgeries were performed by senior surgeons (associate chief physician or above) following standardized protocols. Among the 81 matched patients, 76 (93.8%) achieved R0 resection and 5 (6.2%) had R1 resection. Due to the retrospective and long-term nature of this study, postoperative complications were not systematically documented in a standardized manner for all patients. No surgery-related death occurred in the final study cohort.

Patients in the dCRT group received intensity-modulated radiation therapy (IMRT) with all involved field irradiation, with target delineation standardized according to the NCCN esophageal cancer guidelines and independently reviewed by two senior radiation oncologists to ensure consistency. The gross target volume (GTV) included the primary esophageal tumor (GTVp) and metastatic lymph nodes (GTVn). GTVp was defined as the esophageal lesion identified through various imaging examinations, while GTVn included metastatic lymph nodes visible on CT, ultrasound, and/or MRI. A lymph node was considered GTVn if its short diameter was ≥10 mm (or ≥5 mm for paraesophageal and tracheoesophageal groove nodes), had a high standard uptake value (SUV) on PET-CT (except for inflammatory lymph nodes), or displayed characteristics such as obvious necrosis, ring enhancement, a degree of enhancement similar to that of the primary tumor, or eccentric calcification. The clinical target volume (CTV) included the GTVp and was expanded 5 to 8 mm in the anterior-posterior and left-right directions, and 20 to 30 mm in the superior-inferior direction, which was defined as CTVp. The planned target volume (PTV) was defined as an expansion of 5 mm outward in the anterior-posterior and left-right directions, and 10 to 20 mm in the superior-inferior direction from the CTVp, resulting in PTVp. PTVn was defined as a uniform expansion of the GTVn by 5 to 8 mm. The prescription dose for 193 patients was 60 Gy (2.0 Gy per fraction, 30 fractions), with 95% of the PTVp/PTVn receiving this dose. The remaining 189 patients received Simultaneous Integrated Boost Intensity Modulated Radiation Therapy (SIB-IMRT), with 95% of the PTVp/PTVn receiving a dose of 54 Gy and the GTVp/GTVn receiving a dose of 60 Gy (1.8 to 2.0 Gy per fraction, 30 fractions).All patients completed the planned radiotherapy dose without dose reduction or interruption due to non-treatment-related reasons.

In the S+CRT group, the original esophageal tumor bed area and the corresponding lymph node drainage area (based on the location of the original tumor) were outlined as the CTV. The CTV was expanded by 1.0-2.0 cm in the superior-inferior direction and 0.5 cm in the anterior-posterior and left-right directions to form the PTV. 95% of the PTV was required to receive a dose of 50-54 Gy, administered at 1.8-2.0 Gy per fraction over 25-28 fractions. For patients with a positive stump, a local dose escalation was applied, increasing the dose to 60 Gy. The outlined target area needed to be adjusted to account for anatomical barriers after external radiation exposure. Postoperative chemoradiotherapy was uniformly initiated within 1-3 months after surgery, with no delay exceeding 3 months to ensure treatment timeliness.

Both groups received platinum-based combination chemotherapy regimens with standardized administration cycles and dose intensity:179 patients (46.9%) received platinum combined with paclitaxel, 71 patients (18.6%) received platinum combined with albumin-paclitaxel, and 132 patients (34.6%) received platinum combined with 5-fluorouracil. The number of chemotherapy cycles ranged from 3 to 6, with a median of 4 cycles. In the S+CRT group, 57 patients (52.8%) received platinum combined with paclitaxel, 24 patients (22.2%) received platinum combined with albumin-paclitaxel, and 27 patients (25.0%) received platinum combined with 5-fluorouracil. The number of chemotherapy cycles ranged from 2 to 6, with a median of 3 cycles. All patients completed at least 2 cycles of chemotherapy to ensure the basic efficacy of systemic treatment.

The selection of chemotherapy regimens and radiotherapy doses was based on the routine clinical practice of our institute. For chemotherapy, paclitaxel plus platinum was preferred for patients with good performance status and low risk of peripheral neuropathy. Albumin-paclitaxel plus platinum was administered to patients at high risk of hypersensitivity or neuropathy.5-fluorouracil plus platinum was used for patients with contraindications to taxane agents.

For radiotherapy in the dCRT group, a standard dose of 60 Gy was prescribed, and simultaneous integrated boost intensity-modulated radiation therapy (SIB-IMRT) was applied for relatively large tumors or bulky lymph nodes. In the S+CRT group, adjuvant radiotherapy was delivered at a dose of 50–54 Gy to the tumor bed and regional lymph node drainage areas. For patients with microscopically positive surgical margins, the dose was escalated to 60 Gy.

### 2.4 Data collection

Data collected included the patient’s demographic characteristics (such as age, gender, family history, personal history, and past history), Eastern Cooperative Oncology Group (ECOG) score, and lesion details (including lesion location, length, maximum diameter, and TNM stage). Additionally, pathological type, histological differentiation, radiotherapy and chemotherapy regimens, chemotherapy cycles, radiotherapy modalities, and dose were recorded. Follow-up data encompassed recurrence/progression sites, timing of recurrence, follow-up duration, and patient survival status.

Continuous variables were categorized based on clinical reasoning or statistical methods. Age was classified into ≤64 years and >64 years, while the ECOG score was divided into 0 and 1. Tumor length was stratified into ≤5.0 cm and >5.0 cm. The central lesion location of EC was categorized into three segments: the upper thoracic (Ut) segment (extending from the thoracic inlet to the tracheal bifurcation, corresponding to 18-23 cm from the incisors), the middle thoracic (Mt) segment (from the middle of the tracheal bifurcation to the gastroesophageal junction, corresponding to 24-32 cm from the incisors), and the lower thoracic (Lt) segment (from the middle of the tracheal bifurcation to the gastroesophageal junction, including the abdominal esophagus, corresponding to 32-40 cm from the incisors). Tumor differentiation was classified into poorly differentiated (Pd), moderately differentiated (Md), and well differentiated (Wd). The T stage was divided into T4a and T4b based on the depth of esophageal tumor invasion and involvement of surrounding structures. Lymph node metastasis was classified into N0, N1, N2 and N3 based on the number of affected lymph nodes. Clinicopathological information was collected from medical records, follow-up data, and pathology reports.

### 2.5 Follow-up and effectiveness evaluation

Post-treatment follow-up primarily included routine physical examinations, hematological tests, imaging studies, and cytological assessments. The main imaging modalities utilized were CT, MRI and/or PET/CT. In cases where superficial lymph nodes metastasis was suspected, fine-needle aspiration cytology was performed. If recurrence in the esophagus, anastomotic site, or remnant stomach was suspected, electronic gastroscopy and pathological examination was conducted for confirmation. The unified efficacy evaluation time point was set at 3 months after the completion of all planned treatments (i.e., 3 months after the end of definitive chemoradiotherapy for the dCRT group, and 3 months after the end of postoperative adjuvant chemoradiotherapy for the S+CRT group), and the treatment response was assessed strictly in accordance with the Response Evaluation Criteria in Solid Tumors (RECIST) v1.1 guidelines. All survival endpoint determinations (including progression, recurrence, and death) and efficacy evaluations were independently completed by two senior radiation oncologists and two senior thoracic surgeons with more than 10 years of clinical experience. In case of inconsistent judgments between the evaluators, a third senior clinical oncologist was invited for joint consultation to reach a unified conclusion, which ensured the accuracy and objectivity of endpoint determination. Follow-up evaluations were scheduled every 3 months during the first 2 years post-treatment, every 6 months for the subsequent 3 years, and annually thereafter. Information on patient mortality was obtained from medical records, follow-up data, or official registration with the Chinese Bureau of Statistics. Treatment response was assessed according to the Response Evaluation Criteria in Solid Tumors (RECIST) v1.1 guidelines.

### 2.6 Definition of study outcomes

The outcomes of this study were overall survival (OS) and progression-free survival (PFS).OS was defined as the time interval from the date of pathological confirmation of esophageal squamous cell carcinoma to death from any cause or the last effective follow-up; patients who survived at the last follow-up were defined as censored cases. PFS was defined as the time interval from the date of completion of all planned treatments to disease progression, local recurrence, distant metastasis, death from any cause, or the last effective follow-up; patients without disease progression or death at the last follow-up were defined as censored cases. Disease progression was categorized into local progression (esophagus, anastomosis, regional lymph nodes) and distant progression (distant lymph nodes, tissues, or organs).The specific criteria for disease progression were based on RECIST v1.1 guidelines: (1) Local progression: new lesions appeared in the primary esophageal tumor site, anastomotic stoma or regional lymph node drainage area; or the sum of the maximum diameters of existing local target lesions increased by ≥20% compared with the baseline, with an absolute increase of ≥5 mm; (2) Distant progression: new lesions were confirmed by imaging or pathology in distant organs (liver, lung, bone, brain, etc.) or distant lymph nodes beyond the regional drainage area; (3) Local recurrence: tumor lesions reappeared in the original esophageal lesion site or anastomotic stoma after achieving complete response or partial response post-treatment, confirmed by gastroscopy and pathology.

### 2.7 Statistical analysis

Categorical variables were compared using the chi-square (χ^2^) test. The median follow-up time was estimated using the inverse Kaplan-Meier method. To control the type I error inflation caused by multiple statistical analyses, we pre-specified the primary and secondary endpoints and adopted corresponding type I error control strategies. The primary endpoints were overall survival (OS) and progression-free survival (PFS) of the post-matching cohort, with a two-sided test level (α) set at 0.05 without correction, as these were the pre-defined core research objectives. The secondary endpoints included OS/PFS of the pre-matching cohort, subgroup survival analysis, univariate/multivariate prognostic analysis, and failure mode analysis. For secondary endpoints, we adopted the Bonferroni correction method for type I error control, with the corrected test level (α’) set at 0.01 (α’ = 0.05/5, corresponding to 5 secondary endpoint categories) to avoid false-positive conclusions. Survival curves were generated using the Kaplan-Meier method and compared using the log-rank test. Univariate and multivariate Cox proportional hazards regression models were applied to analyze prognostic factors for OS and PFS. To control for the impact of treatment-related heterogeneity on survival outcomes, the multivariate Cox regression model included not only clinical and pathological covariates but also key treatment-related variables, including chemotherapy regimen (platinum + paclitaxel, platinum + albumin-paclitaxel, platinum + 5-fluorouracil), radiotherapy dose (50-54 Gy, 54-60 Gy, 60 Gy), and surgical approach (VATS, three-incision surgery).

For the post-matching cohort, a stratified Cox proportional hazards regression model was applied to further analyze the independent prognostic value of treatment modality and adjust for residual confounding, with T stage (T4a vs T4b) and N stage (N0 vs N1-N3) as stratified factors. The core principle of the stratified Cox model is to stratify the entire cohort according to the stratified factors with strong prognostic effects; within each stratum, the Cox model is used to analyze the association between the exposure factor (treatment modality) and the outcome (OS/PFS), and the results of each stratum are combined to obtain the overall HR and 95% CI. This method can effectively control the confounding effect of stratified factors on the outcome, and better meet the proportional hazards assumption of the Cox model compared with the traditional multivariate Cox model. All variables in the univariate analysis were included in the multivariate Cox regression model to evaluate their independent predictive value. The results were presented as hazard ratios (HRs) with 95% confidence intervals (CIs) and corresponding P values. A two-sided P-value of <0.05 was considered statistically significant.

Propensity score matching (PSM) was used to reduce selection bias caused by baseline differences between the S+CRT and dCRT groups; its core principle is to calculate a propensity score (PS) for each patient, which is the probability of being assigned to the S+CRT group (versus dCRT group) based on baseline covariates. Patients in the two groups with similar propensity scores have balanced baseline characteristics, which simulates the balance effect of randomized controlled trials and reduces confounding bias. The propensity score was calculated using a logistic regression model, with treatment group (S+CRT vs. dCRT) as the dependent variable, and 8 core baseline clinicopathological covariates as independent variables: age, gender, ECOG performance status, T stage (T4a vs T4b), N stage (N0 vs N1-N3), tumor length, tumor differentiation grade, and primary lesion location. We adopted 1:1 nearest neighbor matching without replacement for PSM implementation; the specific rule is that each patient in the S+CRT group is matched with one patient in the dCRT group with the closest propensity score, and the matched dCRT patient is no longer used for subsequent matching, which ensures the uniqueness of each matched pair and reduces over-matching bias. The caliper width was set at 0.05 times the standard deviation of the logit-transformed propensity scores, which is a widely recognized and validated setting in oncology retrospective studies—this width balances the matching efficiency and balance effect, avoiding overly strict caliper (low matching efficiency, small sample size) or overly loose caliper (poor baseline balance, residual confounding). The balance of baseline characteristics between the two groups was assessed by calculating the absolute standardized mean difference (SMD) for all covariates. After matching, an SMD of less than 0.1 was considered indicative of an adequate balance between the groups, demonstrating the effectiveness of the PSM.

A notable imbalance in sample size existed between the S+CRT and dCRT groups before PSM, which is rooted in clinical practical decision-making for T4N0-3M0 ESCC. Clinically, only T4a-stage patients with good physical status (ECOG 0-1), no severe comorbidities, and resectable tumors are recommended for surgical treatment combined with adjuvant chemoradiotherapy; in contrast, most T4-stage patients (especially T4b-stage) are prioritized for definitive chemoradiotherapy due to high surgical difficulty, high perioperative risk, and low possibility of R0 resection. This sample size discrepancy is an objective reflection of real-world clinical practice for advanced ESCC rather than a methodological flaw. All statistical analyses were performed using R statistical software (version 4.3.2; R Foundation for Statistical Computing, Vienna, Austria; available at http://www.r-project.org).

## 3 Results

### 3.1 Patients’ characteristics

**Figure 1:**
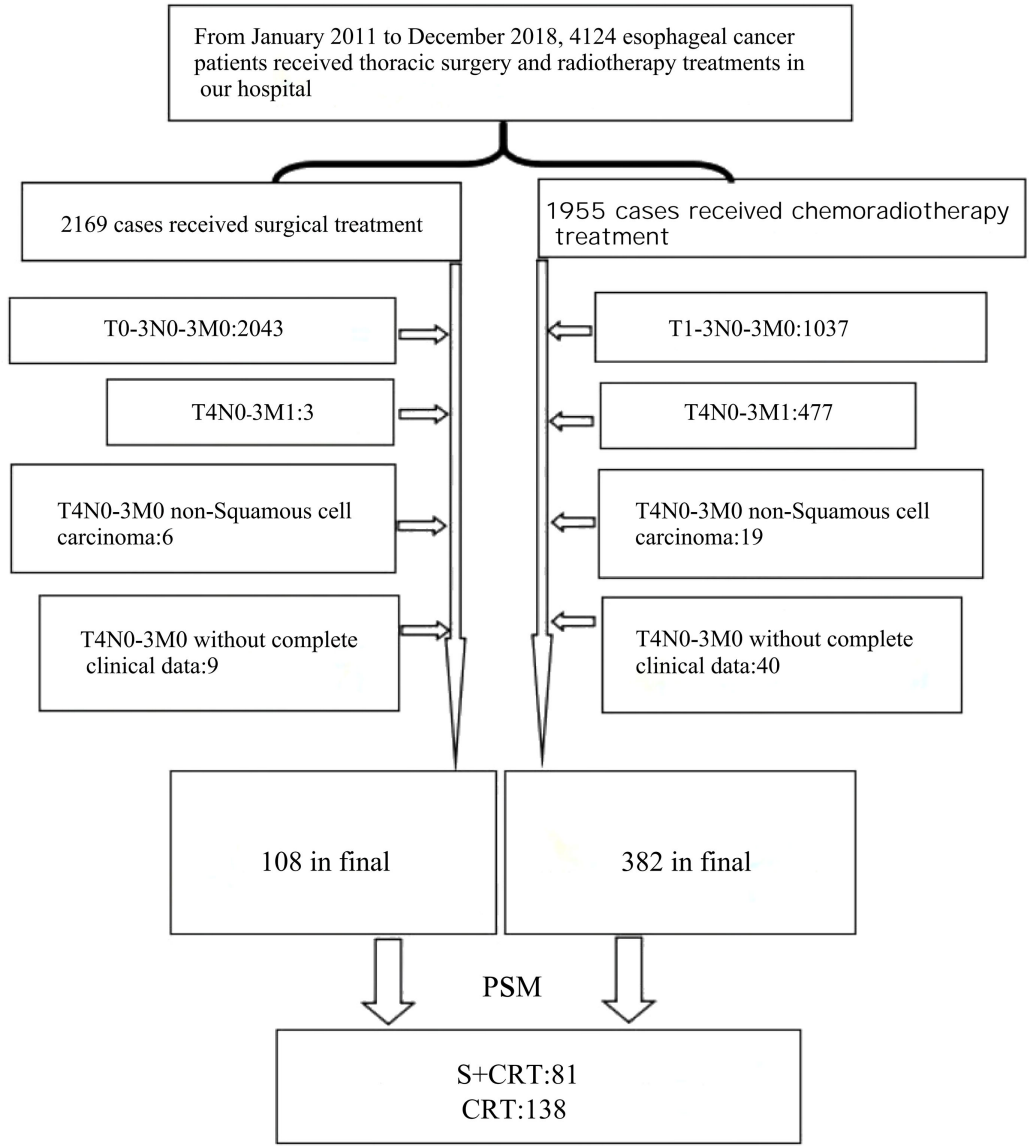
Patient enrollment flow chart.

From January 2011 to December 2018, a total of 4,124 EC patients were identified. Among these, 2,169 patients underwent surgical treatment, while 1,955 patients received chemoradiotherapy. Based on the inclusion and exclusion criteria of this study, 108 patients with stage T4N0-3M0 ESCC who underwent S+CRT and 382 patients with stage T4N0-3M0 ESCC who received dCRT were included. (**Figure 1**).

PSM analysis (maximum random matching) was performed based on the clinicopathological data of the two groups. Among the 382 patients in the dCRT group, 138 were successfully matched, and 244 were not. In the 108 patients of the S+CRT group, 81 were successfully matched, and 27 were not. Baseline characteristics of excluded patients showed that the 27 unmatched S+CRT patients had a higher proportion of T4b stage (62.9% vs. 12.3% in matched S+CRT patients), ECOG score 1 (74.1% vs. 34.6%), and tumor length >5cm (66.7% vs. 28.4%). The 244 unmatched dCRT patients had a higher proportion of T4b stage (78.3% vs. 16.7% in matched dCRT patients), ECOG score 1 (69.3% vs. 32.6%), and poorly differentiated tumors (59.0% vs. 36.2%). After PSM, the absolute SMD for all characteristics were <0.1 (**Table 1**).

**Table 1:** Characteristics before and after PSM in 490 patients with ESCC.

Characteristics	Before PSM			After PSM		
	S+CRT no. (%) N=108	CRT no. (%) N=382	SMD	S+CRT no. (%) N=81	CRT no. (%) N=138	SMD
Gender			0.259			0.011
Male	84 (77.8)	253 (66.2)		62 (76.5)	105 (76.1)	
Female	24 (22.2)	129 (33.8)		19 (23.5)	33 (23.8)	
Age (years)			0.328			0.005
≤64	68(63.0)	179 (46.9)		45 (55.6)	77 (55.8)	
>64	40 (37.0)	203 (53.1)		36 (44.4)	61 (44.2)	
ECOG			0.285			0.039
0	68 (63.0)	187 (49.0)		46 (56.8)	81 (58.7)	
1	40 (37.0)	195 (51.0)		35 (43.2)	57 (41.3)	
Length (cm)			0.625			0.084
≤5.0	44 (40.7)	54 (14.1)		26 (32.1)	39 (28.3)	
>5.0	64 (59.3)	328 (85.9)		55 (67.9)	99 (71.7)	
Location			0.743			0.095
Ut	9 (8.3)	110 (28.8)		9 (11.1)	19 (13.8)	
Mt	49 (45.4)	203 (53.1)		39 (48.1)	70 (50.7)	
Lt	50 (46.3)	69 (18.1)		33 (40.7)	49 (35.5)	
Differentiation			0.426			0.043
Pd	46 (42.6)	157 (41.1)		34 (42.0)	55 (39.9)	
Md+Ud	62 (57.4)	225 (58.9)		47 (58.0)	83 (60.1)	
T Stage			0.556			0.008
T4a	92 (85.2)	235 (61.5)		66 (81.5)	112 (81.2)	
T4b	16 (14.8)	147 (38.5)		15 (18.5)	26 (18.8)	
N Stage			0.127			0.042
N0	49 (45.4)	156 (40.8)		34 (42.0)	60 (43.5)	
N1	47 (43.5)	169 (44.1)		36 (44.4)	61 (44.2)	
N2 + 3	12 (11.1)	57 (14.9)		11 (13.6)	17 (12.3)	

SMD, standardized mean difference; Ut, upper thoracic; Mt, middle thoracic; Lt, lower thoracic; Pd, poorly differentiated; Md, moderately differentiated; Wd, well differentiated.

### 3.2 Analysis of the effectiveness of 219 patients with ESCC after PSM

Among the 81 patients in the S+CRT group, 67 (82.7%) underwent three-incision EC resection, while 14 (12.3%) underwent VATS. Postoperative pathology revealed a positive stump in 11 cases (13.6%) and nerve or vessel invasion in 7 cases (8.6%). Of the total, 76 cases were R0 resections, and 5 were R1 resections. All patients received POCRT after surgery, with radiotherapy performed within 30 to 45 days postoperatively, alongside concurrent chemotherapy. In the dCRT group of 138 patients, 11 (8.0%) achieved a complete response (CR), 110 (79.7%) had a partial response (PR), and 17 (12.3%) showed no change (NC).

### 3.3 Univariate analysis of prognostic factors

**Figure 2:**
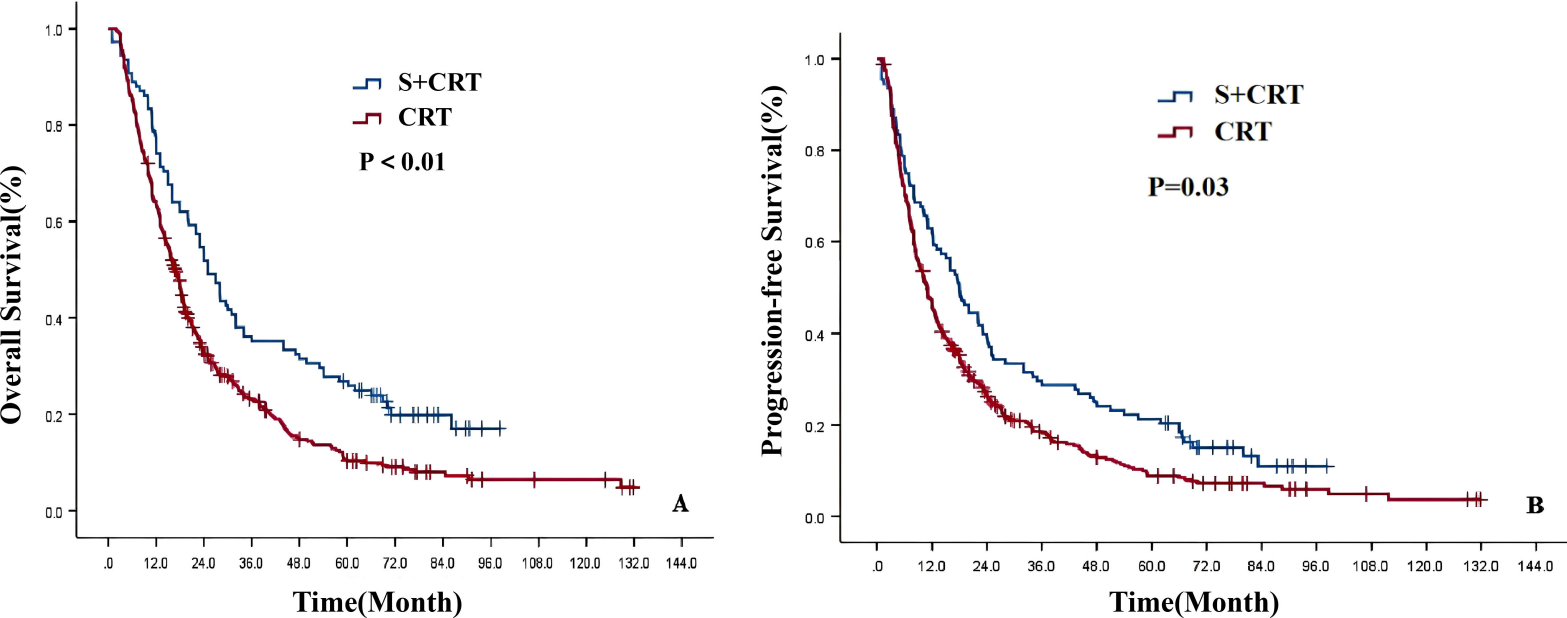
Survival curves of the two treatment modes before PSM [**(A)** OS; **(B)** PFS].

The median follow-up time was 76.5 months (95%CI: 69.8-83.2). The 1-, 3-, 5-, and 8-year OS and PFS rates for the entire cohort of 490 ESCC patients were 65.5%, 25.6%, 14.5%, 9.0%, and 49.3%, 20.6%, 12.0%, and 7.1%, respectively. The median OS and PFS were 18.0 months (95%CI: 16.26-19.74) and 12.0 months (95%CI: 10.54-13.46), respectively. Univariate analysis revealed that the OS and PFS of patients in the S+CRT group were significantly better than those in the dCRT group (χ2 = 15.368, 8.981, P = 0.000, 0.003; secondary endpoint, Bonferroni corrected, P<0.01, statistically significant) (**Supplementary data 1**; **Figures 2A, B**).

**Figure 3:**
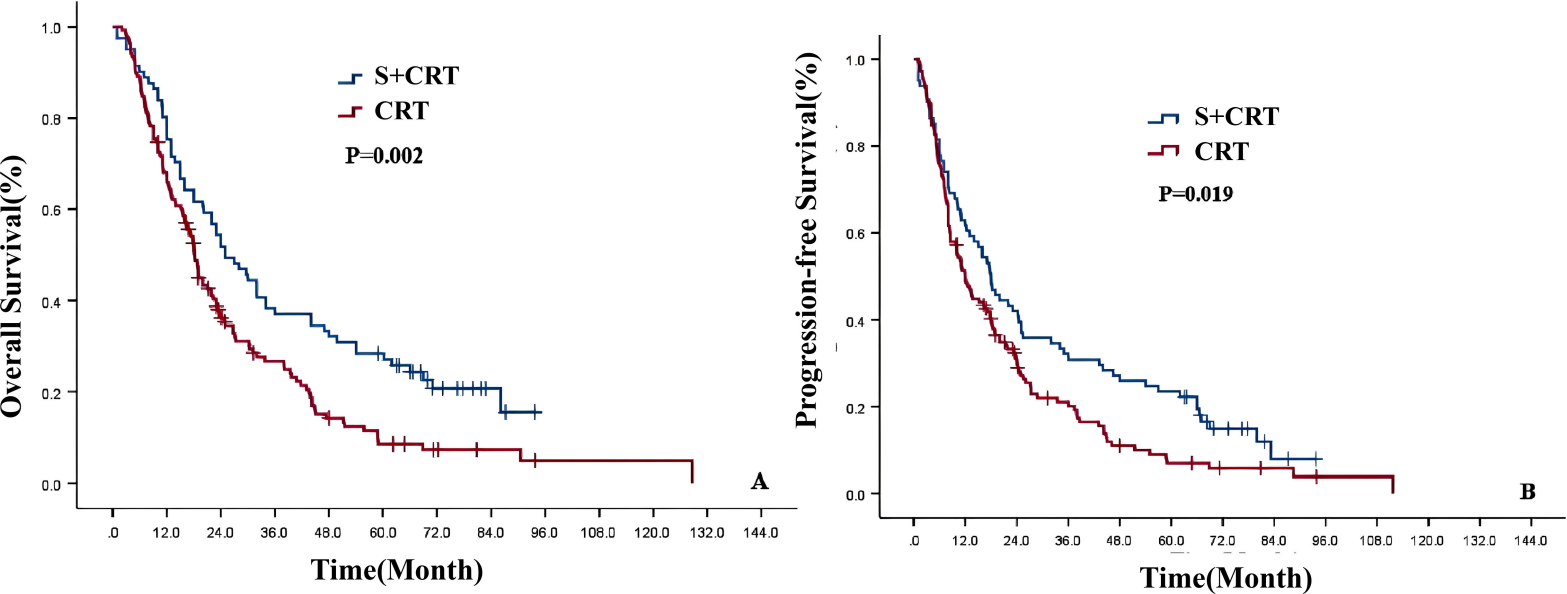
Survival curves of the two treatment modes after PSM [**(A)** OS; **(B)** PFS].

The 1-, 3-, 5-, and 8-year OS and PFS rates for 219 patients with ESCC after PSM were 69.4%, 30.5%, 16.7%, 8.8%, and 53.8%, 24.3%, 13.8%, and 5.3%, respectively. The median OS and PFS were 20.0 months (95%CI: 16.81-23.19) and 14.0 months (95%CI: 10.54-17.47), respectively. Univariate analysis showed that the OS and PFS of patients in the S+CRT group were significantly better than those in the dCRT group (χ2 = 9.488, 5.484, P = 0.002, 0.019;primary endpoint, no correction, P<0.05,statistically significant) (**Supplementary data 1**; **Figures 3A, B**).

**Figure 4:**
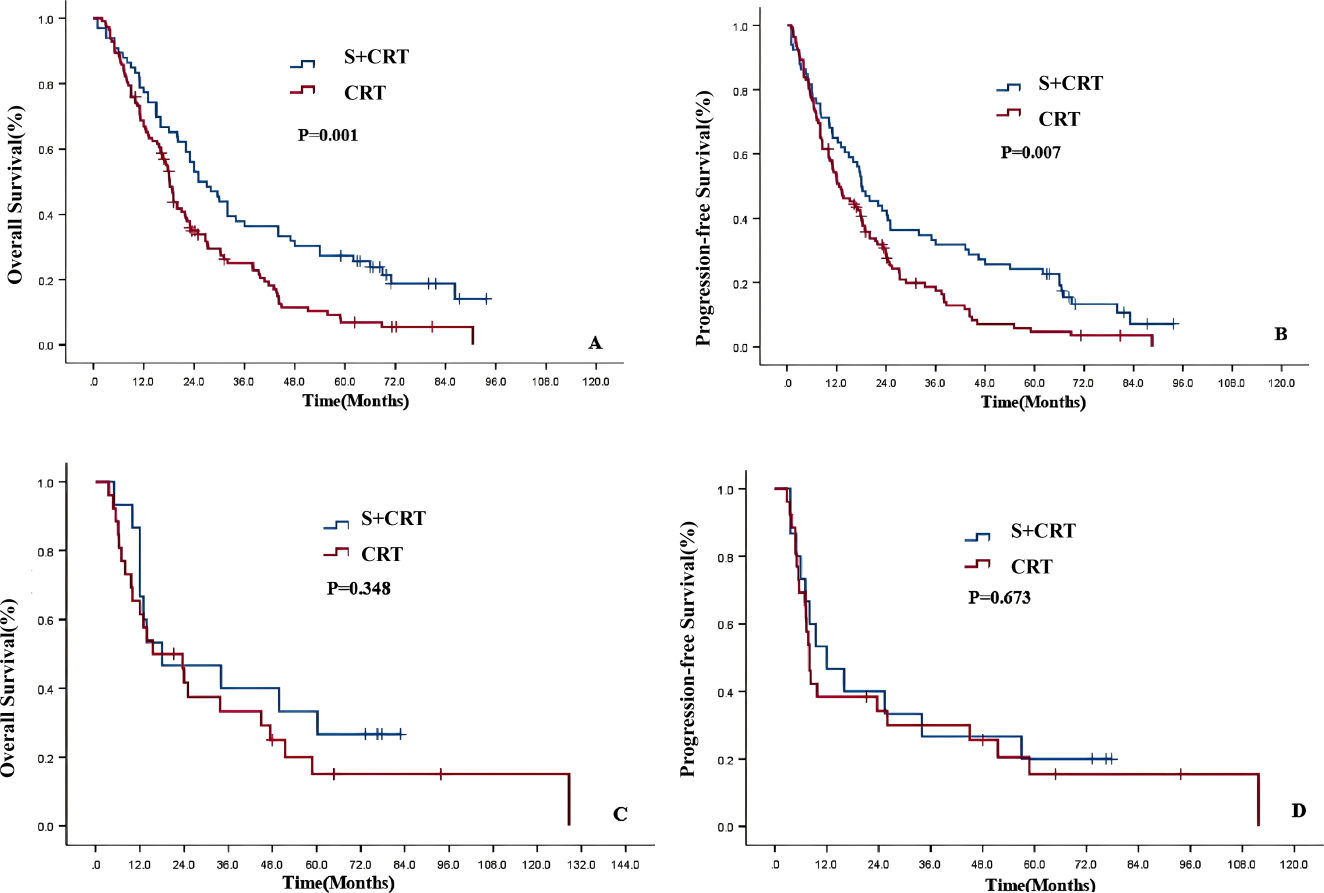
Survival curve of different T stages on prognosis of the two treatment modes after PSM [**(A)** OS of T4a; **(B)** PFS of T4a; **(C)** OS of T4b; **(D)** PFS of T4b].

Univariate analysis was further performed on 219 ESCC patients who received different treatments based on T stage (**Supplementary data 2**; **Figure 4**). For T4a stage ESCC patients, those who received S+CRT had better OS (P = 0.001;secondary endpoint, Bonferroni corrected, P<0.01, statistically significant) and PFS (P = 0.007;secondary endpoint, Bonferroni corrected, P<0.01, statistically significant**) compared to the dCRT group (**Supplementary data 2**; **Figures 4A, B**). For patients with stage T4b stage ESCC, there was no significant difference in OS (P = 0.348; **secondary endpoint, Bonferroni corrected, P>0.01, no statistical significance) or PFS (P = 0.673;secondary endpoint, Bonferroni corrected, P>0.01, no statistical significance) between the S+CRT and dCRT groups ***(*****Supplementary data 2**; **Figures 4C, D*****)***.

### 3.4 Prognostic multivariate analysis results

The results of the COX multivariate analysis for 490 ESCC patients (before PSM) revealed that N stage and treatment modality were independent prognostic factors for OS (HR = 1.33,1.48; 95%CI:1.15-1.53,1.12-1.97;P=0.000,0.006; secondary endpoint, Bonferroni corrected, P<0.01,statistically significant) and PFS (HR = 1.42, 1.37; 95%CI: 1.24-1.63, 1.04-1.79; P=0.000, 0.024; secondary endpoint, Bonferroni corrected, OS P<0.01,statistically significant; PFS P>0.01, no statistical significance) (**Supplementary data 3**).

The results of the COX multivariate analysis for 219 ESCC patients (after PSM) showed that N stage and treatment modality were independent prognostic factors for OS (HR = 1.54, 1.64; 95%CI: 1.23-1.93, 1.18-2.27; P=0.000, 0.003; primary endpoint, no correction, P<0.05, statistically significant) and PFS (HR = 1.59, 1.48; 95%CI: 1.28-1.97, 1.08-2.04; P=0.000, 0.0155; primary endpoint, no correction, P<0.05, statistically significant) (**Supplementary data 4**).

**Figure 5:**
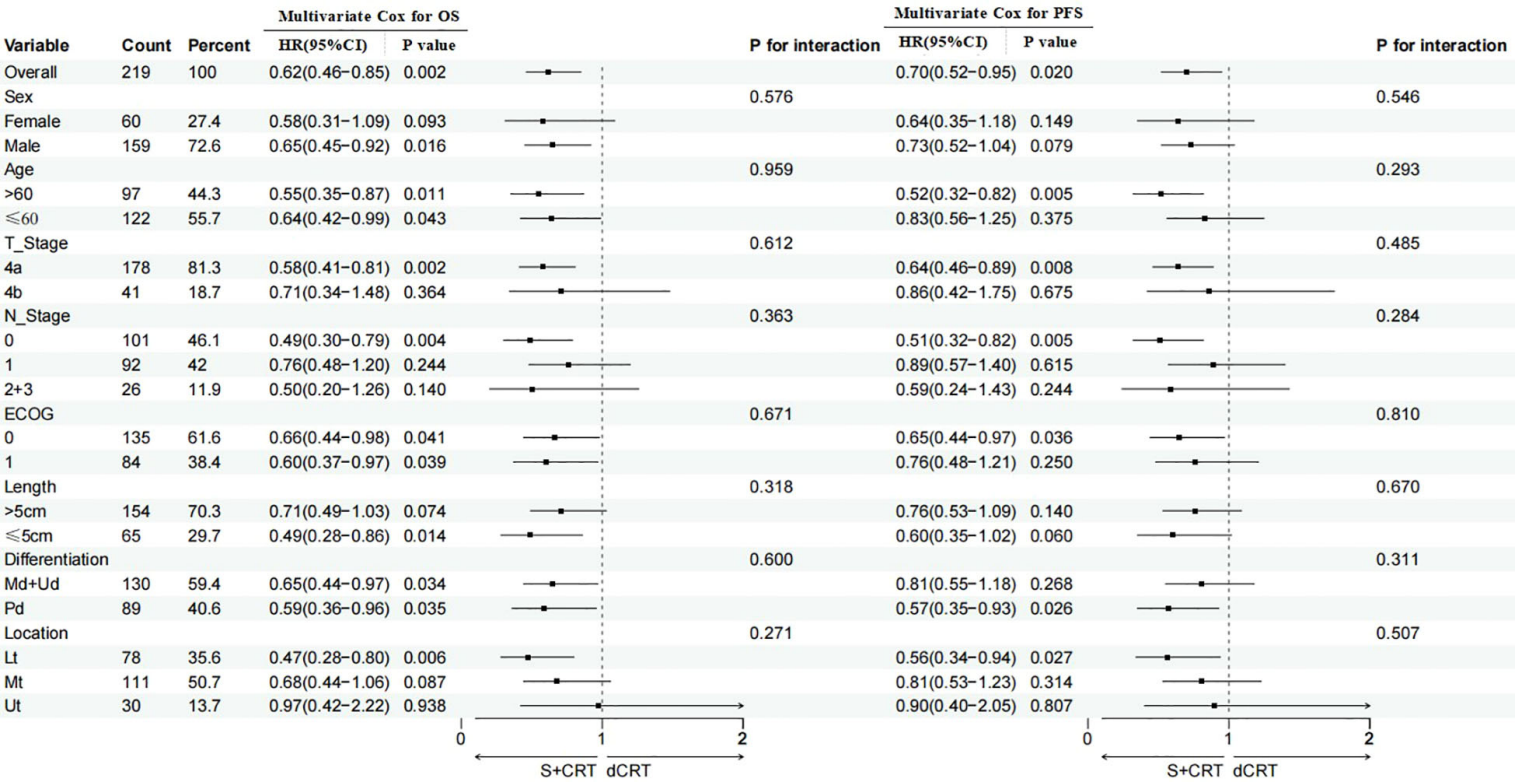
Subgroup analysis of 219 patients with ESCC.

Stratified Cox proportional hazards regression analysis for the post-matching cohort (stratified by T stage and N stage) showed that treatment modality remained an independent protective factor for OS (HR = 1.69, 95%CI: 1.22-2.34; P = 0.002; primary endpoint, no correction, P<0.05, statistically significant) and PFS (HR = 1.52, 95%CI: 1.11-2.09; P = 0.009; primary endpoint, no correction, P<0.05, statistically significant), after fully controlling for the confounding effects of T stage and N stage. Stratified subgroup results showed that in the T4a stratum, S+CRT was associated with better OS (HR = 1.72, 95%CI: 1.19-2.48; P = 0.004) and PFS (HR = 1.56, 95%CI: 1.07-2.28; P = 0.021); in the T4b stratum, no significant difference was found in OS (HR = 1.31, 95%CI: 0.72-2.39; P = 0.376) or PFS (HR = 1.25, 95%CI: 0.68-2.29; P = 0.471). In the N0 stratum, S+CRT significantly improved OS (HR = 1.81, 95%CI: 1.15-2.85; P = 0.010) and PFS (HR = 1.63, 95%CI: 1.02-2.60; P = 0.042); in the N1-N3 stratum, the survival benefit of S+CRT was not statistically significant (OS: HR = 1.45, 95%CI: 0.89-2.36; P = 0.137; PFS: HR = 1.32, 95%CI: 0.81-2.16; P = 0.258) (**Supplementary data 5**; **Figure 5**). All results of the stratified Cox model were consistent with the core conclusion that S+CRT has a survival advantage over dCRT in the post-matching cohort.

### 3.5 Subgroup analysis of 219 ESCC receiving two treatment modalities after PSM

Note: This subgroup analysis is exploratory in nature, and the results need to be interpreted with caution. In the subgroup analysis, the OS of the following subgroups benefited from the S+CRT treatment model: male, any age, T4a stage, N0 stage, ECOG score 0/1, lesion length ≤ 5.0 cm, any degree of tissue differentiation, and Lt segment. In contrast, the PFS was improved in the subgroups of patients aged >60 years, T4a stage, N0 stage, ECOG score 0, tissue differentiation of Pd, and Lt segment who received the S+CRT treatment model (secondary endpoint, Bonferroni corrected, all P<0.01, statistically significant) (**Figure 5**).

### 3.6 Failure mode analysis results

Comparing the failure modes of 219 patients after PSM, at the time of follow-up, 41 patients (50.6%) in the S+CRT group experienced local recurrence or distant metastasis, while 73 patients (52.9%) in the dCRT group had similar outcomes. The overall failure patterns of the two groups were significantly different (χ^2^ = 9.553, P = 0.023; secondary endpoint, Bonferroni corrected, P>0.01, no statistical significance) (**Supplementary data 5**). Further analysis revealed that the incidence of distant metastasis was slightly higher in the S+CRT group (34.5%) compared to the dCRT group (20.0%), while the overall local recurrence rate was lower in the S+CRT group (28.3%) than in the dCRT group (37.0%).

In the S+CRT group, 16 patients received salvage radiotherapy, and 21 patients received salvage chemotherapy. In the dCRT group, 11 patients received salvage radiotherapy, 3 patients underwent salvage surgery, and 32 patients received salvage chemotherapy.

## 4 Discussion

Clinical T4 stage EC accounts for approximately 15% of all newly diagnosed cases (12). For patients with this stage of EC, treatment approaches vary significantly across different centers, and no clinical consensus has been established, the overall treatment outcomes remain unsatisfactory (13). Currently, three primary treatment strategies are commonly employed in clinical practice (11, 14–16): preoperative neoadjuvant chemotherapy (or chemoradiotherapy) followed by surgery, postoperative adjuvant radiotherapy (or chemoradiotherapy), and dCRT. With the advent of immune checkpoint inhibitors (ICIs), immunotherapy-based combination treatments have emerged as a promising approach for advanced EC. However, challenges remain, and long-term survival data are currently lacking (17). Numerous studies have demonstrated that patients who achieve a favorable therapeutic response following neoadjuvant therapy benefit from subsequent surgical treatment, whereas those with a poor response show no significant advantage in effectiveness compared with dCRT (5–8). Although POCRT for EC lacks robust evidence-based support and is seldom recommended by major guidelines, several studies have reported that POCRT can enhance prognosis in certain postoperative ESCC patients. In particular, it has been shown to significantly improve survival in patients with positive lymph nodes and deep local invasion (9–11). With the widespread adoption of IMRT in clinical practice, concurrent chemotherapy can now be administered more safely. Additionally, organ preservation has become a key focus in EC management, especially when achieving an R0 resection is uncertain. In such cases, surgical intervention is not the preferred option, leading most clinical T4 patients to receive dCRT as the primary treatment approach. Currently, the role of surgical treatment alone in stage T4 ESCC remains unclear. Previous surgery-related studies have primarily focused on salvage surgery following dCRT (18, 19) and the combination of preoperative neoadjuvant therapy with surgery. Based on our literature review, this study represents the largest to date, with a substantial sample size and a follow-up period exceeding five years. It provides a comparative analysis of two distinct treatment strategies—S+CRT and dCRT—for patients with T4 ESCC.

This study demonstrated that, compared with dCRT, the S+CRT treatment model provided significant advantages for patients with stage T4 ESCC. Multivariate analysis conducted both before and after PSM consistently identified the S+CRT treatment model as an independent factor associated with improved long-term OS and PFS.

Previous studies on POCRT have demonstrated that postoperative EC patients with locally advanced and positive lymph nodes benefit more from surgery combined with POCRT than from surgery alone. Some studies have further indicated that POCRT provides superior survival benefits compared with postoperative adjuvant chemotherapy alone (20, 21). Qi et al. (5) investigated whether patients with T4-stage EC could benefit from surgical treatment. Their study included a total of 1,822 patients with T4N0-3M0 disease. The results indicated that in all population cohorts (T4N0-3M0, T4aN0-3M0, and T4bN0-3M0), the addition of surgery significantly improved patient prognosis. Specifically, for patients undergoing surgery alone without neoadjuvant therapy, the HR for OS compared to those who did not undergo surgery was 0.464 (95%CI: 0.375-0.574, P<0.001). However, no significant difference in OS was observed between patients receiving neoadjuvant therapy plus surgery and those undergoing surgery alone without neoadjuvant therapy (HR = 0.966, 95%CI: 0.686-1.360, P = 0.843). The study concluded that the addition of surgery could improve OS in patients with T4N0-3M0 EC, regardless of whether they had T4a or T4b disease, and that the benefit of surgery was independent of the use of neoadjuvant therapy. The findings of these studies align with the results of the present study, indicating that the addition of surgical treatment provides a survival benefit for patients with stage T4 ESCC. Notably, even though the patients in this study did not receive neoadjuvant therapy but instead underwent POCRT, this treatment approach still yielded significant benefits. This survival advantage may be attributed to the ability of POCRT to effectively control the growth of subclinical lesions, eradicate residual tumor cells, prevent metastasis, enhance surgical success rates, and reduce the likelihood of tumor recurrence.

For patients with a confirmed diagnosis of stage T4b ESCC, the standard recommended treatment is dCRT (22). However, despite advancements in imaging diagnostics and other technologies, the accuracy of preoperative staging remains suboptimal. Many cases initially considered surgically resectable are later found to be unresectable during surgery and are ultimately diagnosed as stage T4b (23, 24). In this study, subgroup analysis of patients with stage T4a and T4b ESCC revealed that patients with stage T4a significantly benefit from the S+CRT treatment approach, whereas for stage T4b patients, no significant prognostic difference was observed between the two treatment modalities. There are two possible reasons for this finding. First, the sample size of stage T4b patients in this study was relatively small. Second, tumors in stage T4b ESCC are often closely associated with surrounding normal tissues and organs, making complete resection challenging. These findings are consistent with the study by Fujii et al. (25), which analyzed 47 patients with stage T4b ESCC. In their study, the 2-year OS rates for 34 patients undergoing palliative surgery and 13 patients who did not undergo surgery were 0% and 20.2%, respectively (P = 0.882), with the incidence of ≥ grade 3 treatment-related complications being 73.5% and 23.1%, respectively. They concluded that palliative esophagectomy should be avoided due to its high complication rate and lack of long-term survival benefits. Therefore, achieving a more accurate preoperative diagnosis is of utmost importance. For ESCC patients whose preoperative assessment suggests that the esophageal lesions are closely associated with adjacent normal tissues and organs, and for whom R0 resection is unlikely to be achieved, direct dCRT or translational therapy should be recommended (26, 27).

Local recurrence and/or distant metastasis are the primary causes of treatment failure in ESCC patients, particularly for those with advanced T-stage disease (28). However, different treatment modalities result in varying failure rates (3, 7, 25, 29). Patients who undergo surgical treatment tend to have a lower regional recurrence rate compared to those who do not receive surgery, likely due to more effective resection of the primary tumor and metastatic lesions. When combined with subsequent radiotherapy and chemotherapy, the risk of micro-metastatic lesions is also reduced to some extent.

This study has several limitations. First, as a retrospective single-center analysis, potential selection bias cannot be fully excluded. For example, patients in the surgical group had a higher proportion of ECOG 0 scores, which may reflect better baseline physical condition. Additionally, resectability likely influenced treatment selection, with a higher proportion of T4a cases observed in the surgical group. To address these issues, we applied propensity score matching to balance key baseline variables, and performed subgroup analyses stratified by T stage to reduce the impact of resectability differences. While these methods help mitigate bias, unmeasured confounders may still exist, such as patients’ nutritional status, comorbidity severity, surgeon’s operative experience, radiation oncologist’s target delineation proficiency, and treatment compliance. These unmeasured factors may lead to residual confounding bias: for instance, patients with better nutritional status and fewer severe comorbidities are more likely to be selected for surgical treatment and tend to have a favorable prognosis independent of treatment modalities, and the heterogeneity of surgeon experience may also affect surgical efficacy and perioperative complication rates. Notably, residual confounding bias cannot be completely eliminated despite our best efforts in statistical adjustment, which may potentially affect the interpretation of results to some extent. We further emphasize that the single-center nature of this study imposes notable restrictions on the external validity and generalizability of the findings. All enrolled patients were pathologically confirmed ESCC treated at a single tertiary cancer center in North China, with consistent diagnostic criteria, treatment protocols and follow-up standards; thus, the results are only applicable to T4N0-3M0 ESCC patients with good organ function, who can tolerate radical surgery or definitive chemoradiotherapy, and are not applicable to patients with esophageal adenocarcinoma, small cell carcinoma, those with severe underlying diseases, poor treatment tolerance, or those receiving treatment at primary or secondary medical institutions with different technical conditions. Due to regional differences in ESCC epidemiology, tumor biological characteristics and clinical treatment preferences, the conclusions cannot be directly extrapolated to ESCC populations in other regions of China or overseas. The matched cohort has good representativeness: all covariates achieved an absolute SMD <0.1 after matching, consistent with the criteria for effective matching. Moreover, the unmatched patients were mainly those with extreme baseline characteristics (high T4b proportion, poor physical status, long tumor length) that deviated from the main population of T4N0-3M0 ESCC, and the core conclusion that S+CRT improves survival outcomes was consistent in both pre-matching full cohort and post-matching cohort analysis. This confirms that the exclusion of unmatched patients does not affect the reliability of the study conclusion. We have implemented multiple measures to control treatment heterogeneity: first, standardized treatment procedures were adopted, including unified surgical methods, standardized radiotherapy target delineation and dose range, and platinum-based chemotherapy regimens with consistent dose intensity; second, key treatment-related variables (chemotherapy regimen, radiotherapy dose, surgical approach) were included in the multivariate Cox regression model for statistical adjustment; third, subgroup analysis by T stage was performed to reduce the interference of treatment heterogeneity in different subgroups. Despite these control measures, potential residual treatment heterogeneity still exists: slight differences in the timing of chemotherapy administration and individual tolerance to treatment may affect survival outcomes, and the lack of unified molecular typing may lead to different treatment responses among patients with the same clinical stage. These residual heterogeneities may have a mild impact on the results, but the consistent survival benefit of S+CRT in both pre- and post-matching cohorts suggests that the core conclusion is not affected. Furthermore, the subgroup analysis in this study has limitations in statistical power: the sample size of some subgroups (e.g., T4b stage, well-differentiated tumors) is relatively small, which may lead to insufficient statistical power to detect potential differences, increasing the risk of false-positive or false-negative results. It is important to emphasize that this study does not draw definitive treatment recommendations based on the results of subgroup analysis; the subgroup findings only provide preliminary clues for subsequent targeted research and cannot guide clinical treatment decisions directly. For further verification and expansion of the conclusions, we plan to launch a multicenter, prospective cohort study in the next phase, which will enroll T4N0-3M0 ESCC patients from multiple tertiary cancer centers in different regions of China. The prospective study will standardize diagnostic criteria, treatment protocols and follow-up procedures, comprehensively collect data on nutritional status, comorbidity severity, molecular markers (such as PD-L1 expression, tumor mutation burden) and other potential confounders, and further explore the efficacy and safety of S+CRT versus dCRT combined with immunotherapy, to provide more high-level evidence-based medicine for the clinical treatment of T4N0-3M0 ESCC. Therefore, the core conclusion of this study needs to be further verified and confirmed in multicenter prospective cohorts with more standardized data collection and stricter confounding control. Second, all patients included in this study were diagnosed with ESCC, and whether the findings are applicable to patients with other pathological types of EC requires further verification. Third, this study primarily focused on comparing the advantages and disadvantages of the two treatment modalities, and there was no standardized chemotherapy regimen for all patients, which could introduce potential bias. Additionally, this study lacked routine detection and analysis of immune or molecular biological markers, such as PD-L1 expression, tumor mutation burden, and gene mutations. During the study period (2011-2018), biomarker testing was not standard clinical practice for locally advanced esophageal squamous cell carcinoma in our center. This represents an important limitation that may restrict further exploration of predictive markers for treatment response. Therefore, further research is needed to explore the gene mutation spectrum, tumor microenvironment, and associated signaling pathways in ESCC. With the advent of immunotherapy, we plan to conduct larger prospective studies to validate our findings and compare ICIs with traditional treatments in T4 ESCC patients.

## 5 Conclusion

For patients with T4a stage, N0 stage, Lt segment, ECOG score 0, and poor differentiation, the S+CRT treatment model presents a promising option, potentially extending long-term survival. However, treatment decisions should be based on a comprehensive evaluation of both the patient’s individual condition and the characteristics of the tumor to ensure a personalized approach.

## Data Availability

The raw data supporting the conclusions of this article will be made available by the authors, without undue reservation.
