# Supplementary material for: Long-term prognostic comparison of surgery followed by adjuvant chemoradiotherapy versus definitive chemoradiotherapy in T4N0-3M0 esophageal squamous cell carcinoma: a single-center retrospective cohort study
Source: Front Oncol. 2026 Mar 24;16:1743644. doi: 10.3389/fonc.2026.1743644 (PMC13053226; doi:10.3389/fonc.2026.1743644)
Supplement: Supplementary file 1 [file Table1.doc]

**Supplementary data 1. Efficacy of two treatment modalities (before and after PSM)**

| Treatment | No. | OS(%) | | | | mOS (months) | χ^2^ | P | PFS(%) | | | | mPFS (months) | χ^2^ | P |
| --- | --- | --- | --- | --- | --- | --- | --- | --- | --- | --- | --- | --- | --- | --- | --- |
|  |  | 1y | 3y | 5y | 8y |  |  |  | 1y | 3y | 5y | 8y |  |  |  |
| **Before PSM** |  |  |  |  |  |  | 15.368 | 0.000 |  |  |  |  |  | 8.981 | 0.003 |
| S+CRT | 108 | 74.1 | 35.2 | 26.9 | 17.0 | 25.0 |  |  | 62.0 | 28.7 | 21.3 | 11.0 | 18.0 |  |  |
| dCRT | 382 | 63.1 | 22.9 | 10.3 | 6.4 | 16.9 |  |  | 45.6 | 18.2 | 8.8 | 5.9 | 10.9 |  |  |
| **After PSM** |  |  |  |  |  |  | 9.488 | 0.002 |  |  |  |  |  | 5.484 | 0.019 |
| S+CRT | 81 | 75.3 | 37.0 | 28.4 | 15.6 | 25.0 |  |  | 61.7 | 30.9 | 23.5 | 7.9 | 18.0 |  |  |
| dCRT | 138 | 65.9 | 26.7 | 8.5 | 4.9 | 18.2 |  |  | 49.2 | 20.1 | 7.0 | 3.9 | 12.0 |  |  |
