# Supplementary material for: Long-term prognostic comparison of surgery followed by adjuvant chemoradiotherapy versus definitive chemoradiotherapy in T4N0-3M0 esophageal squamous cell carcinoma: a single-center retrospective cohort study
Source: Front Oncol. 2026 Mar 24;16:1743644. doi: 10.3389/fonc.2026.1743644 (PMC13053226; doi:10.3389/fonc.2026.1743644)
Supplement: Supplementary file 2 [file Table2.doc]

**Supplementary data 2. Influences of different T stages on the efficacy of two treatment modalities (after PSM)**

| Treatment | No. | OS(%) | | | | mOS (months) | χ^2^ | P | PFS(%) | | | | mPFS (months) | χ^2^ | P | |
| --- | --- | --- | --- | --- | --- | --- | --- | --- | --- | --- | --- | --- | --- | --- | --- | --- |
|  |  | 1y | 3y | 5y | 8y |  |  |  | 1y | 3y | 5y | 8y |  |  |  |  |
| **T4a** |  |  |  |  |  |  | 10.198 | 0.001 |  |  |  |  |  | 7.197 | | 0.007 |
| S+CRT | 66 | 77.3 | 36.4 | 27.3 | 14.1 | 25.0 |  |  | 65.2 | 31.8 | 24.2 | 7.1 | 18.0 |  | |  |
| dCRT | 112 | 66.9 | 25.1 | 6.9 | 0.0 | 18.2 |  |  | 51.6 | 17.5 | 4.7 | 0.0 | 12.6 |  |  | |
| **T4b** |  |  |  |  |  |  | 0.880 | 0.348 |  |  |  |  |  | 0.178 | 0.673 | |
| S+CRT | 15 | 66.7 | 40.0 | 26.7 | 26.7 | 18.0 |  |  | 46.7 | 26.7 | 20.0 | 20.0 | 12.0 |  |  | |
| dCRT | 26 | 61.5 | 33.3 | 15.0 | 15.0 | 15.5 |  |  | 38.5 | 29.9 | 15.4 | 15.4 | 8.0 |  |  | |
