# Supplementary figures and images for: Long-term prognostic comparison of surgery followed by adjuvant chemoradiotherapy versus definitive chemoradiotherapy in T4N0-3M0 esophageal squamous cell carcinoma: a single-center retrospective cohort study
Source: Front Oncol. 2026 Mar 24;16:1743644. doi: 10.3389/fonc.2026.1743644 (PMC13053226; doi:10.3389/fonc.2026.1743644)

Supplementary data 3. COX multivariate analysis results of 490 patients with ESCC (before PSM)


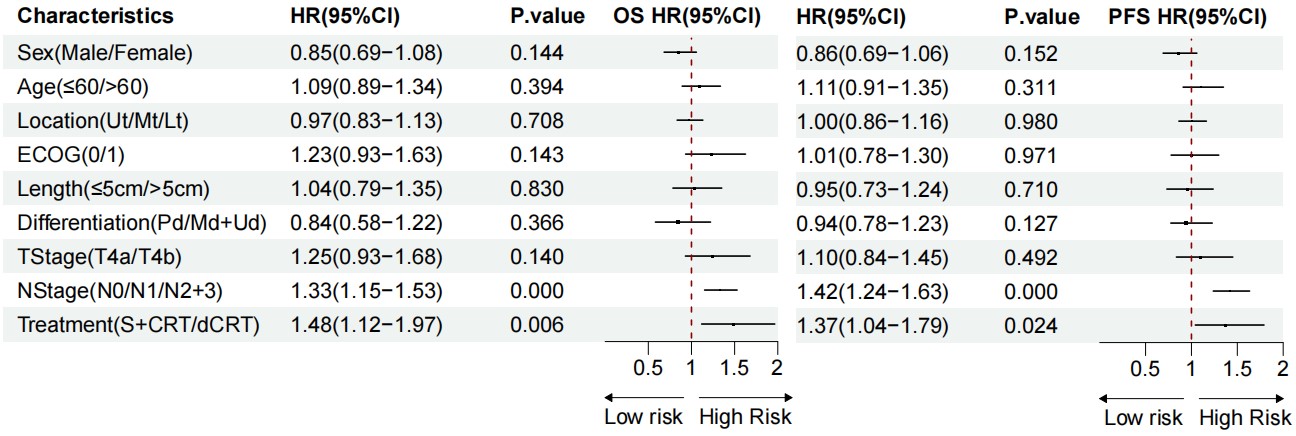

Supplement: Supplementary file 3 [file Table3.doc]

Supplementary data 4. COX multivariate analysis results of 219 patients with ESCC (after PSM)


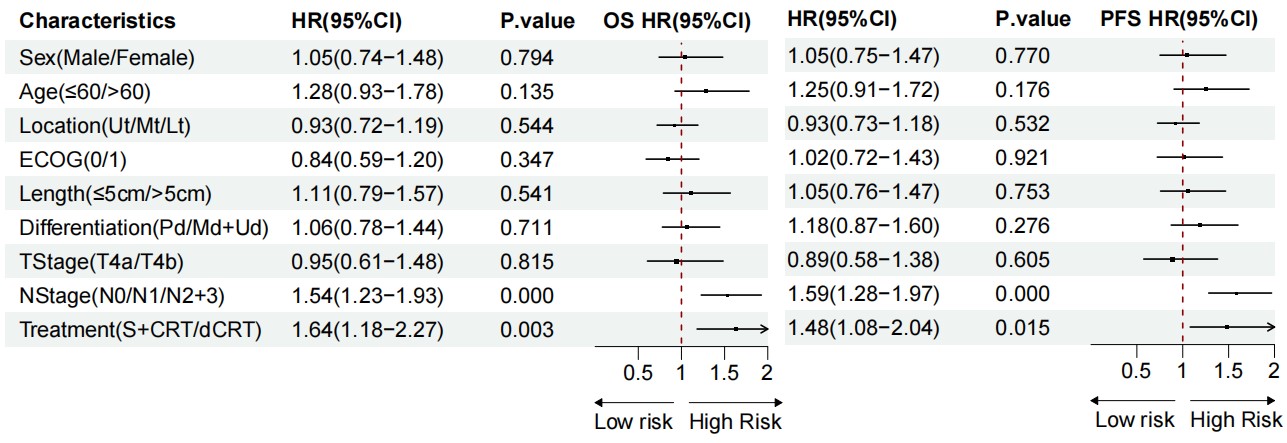

Supplement: Supplementary file 4 [file Table4.doc]
