# Supplementary material for: Long-term prognostic comparison of surgery followed by adjuvant chemoradiotherapy versus definitive chemoradiotherapy in T4N0-3M0 esophageal squamous cell carcinoma: a single-center retrospective cohort study
Source: Front Oncol. 2026 Mar 24;16:1743644. doi: 10.3389/fonc.2026.1743644 (PMC13053226; doi:10.3389/fonc.2026.1743644)
Supplement: Supplementary file 5 [file Table5.doc]

**Supplementary data 5. Failure patterns of 219 ESCC patients with two treatment modalities**

| Treatment modalities | Failure patterns No.(%) | | | |
| --- | --- | --- | --- | --- |
|  | None | Local recurrence | Distant metastasis | Both |
| S+CRT | 40（49.4） | 13（16.0） | 18（22.2） | 10（12.3） |
| dCRT | 65（47.1） | 44（31.9） | 22（15.9） | 7（5.1） |
| χ^2^ | 9.553 | | | |
| P | 0.023 | | | |
